# Removal of a migrated dental implant from a maxillary sinus through an intraoral approach: A case report

**DOI:** 10.4317/jced.58350

**Published:** 2021-07-01

**Authors:** Enrique Núñez-Márquez, Angel-Orión Salgado-Peralvo, Juan-Francisco Peña-Cardelles, Naresh Kewalramani, Alvaro Jiménez-Guerra, Eugenio Velasco-Ortega

**Affiliations:** 1 Professor of Master in Implant Dentistry, University of Seville, US, Seville, Spain; 2 Master in Implant Dentistry, US. Master’s Degree in Family and Community Dentistry, US; 3 Professor of the Postgraduate Program in Oral Surgery and Implantology. Rey Juan Carlos University, URJC, Madrid, Spain; 4 Professor of the Postgraduate Program in Oral Surgery, Periodontology and Implantology, URJC; 5 Professor of Comprehensive Dentistry for Adutls and Gerodontology, US. Director of the Postgraduate Program in Implant Dentistry, US

## Abstract

**Background:**

The replacement of maxillary posterior teeth often challenges the clinician due to bone resorption after dental exodontia and low bone quality. Currently, attempts are being made to shorten treatment times by placing implants simultaneously to sinus lift procedures in borderline cases, which can lead to complications such as displacement of implants into the maxillary sinus.

**Clinical Case:**

A 54-year-old patient who was planned for complete rehabilitation of the maxilla through a fixed implant-supported prosthesis on 6 implants. At the level of the 3rd sextant, a sinus lift was performed with a lateral window approach (Caldwell-Luc type) and the simultaneous placement of two implants, one of which migrated into the sinus. The implant was displaced after 4 months when the second stage (uncovering) implant surgery was performed for the connection of the healing abutments. The implant was removed a week after the migration, since it had moved to the tuberosity area in the sinus and the lateral window had been performed in a more mesial position, so the patient was recommended to sleep on the right side to achieve the displacement of the implant to a more favourable area, removing it after a week through the same approach.

**Discussion:**

Surgical strategies for the removal of a migrated implant are essentially divided into two main approaches: endoscopic transoral and endoscopic transnasal (and combined).

**Conclusions:**

In case of intra-operative migration of the implant into the sinus, it is recommended to remove it as soon as possible to avoid a possible sinus pathology of iatrogenic origin.

** Key words:**Dental implant complications, dental implant, dental implant displacement, maxillary sinus, case report.

## Introduction

Rehabilitation through dental implants of the posterior maxilla is often a challenge for the oral and maxillofacial surgeon for the following reasons: a) resorption of the alveolar ridge; b) progressive pneumatization of the maxillary sinus and (c) low density of the alveolar bone (8). These constraints make it difficult to place implants and can cause complications, such as implant displacement into the maxillary sinus (DID) (3). Dental implant displacement (DID) is a relatively unusual complication, but one survey by the Japanese Academy of Maxillofacial Implants on perioperative complications revealed that, among a total of 421 cases, 63 (15%) involved the migration of the implant to the maxillary sinus (4).

This article aims to show and describe the procedure of the removal of an implant displaced to the maxillary sinus through an intra-oral approach.

Case Report

**Figure 1:**
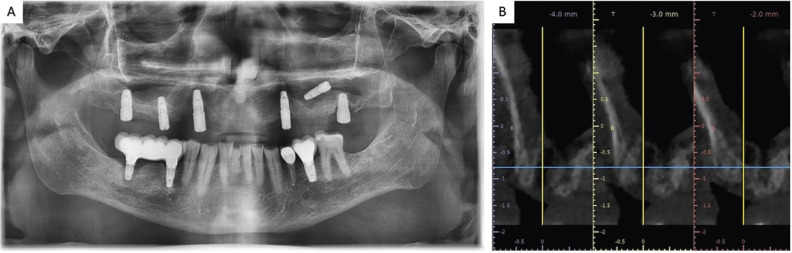
A. Orthopantomography performed intraoperatively, after migration of the implant into the sinus cavity. B. Cone-beam computerized tomography performed before the rescue of the migrated implant.

**Figure 2:**
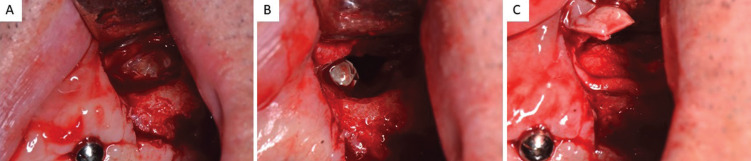
A. Access to lateral antrostomy performed one week before when the implant migrated into the sinus. B. Perforation of the Schneider membrane and location of the implant in the sinus cavity. C. Removal of the implant.

**Figure 3:**
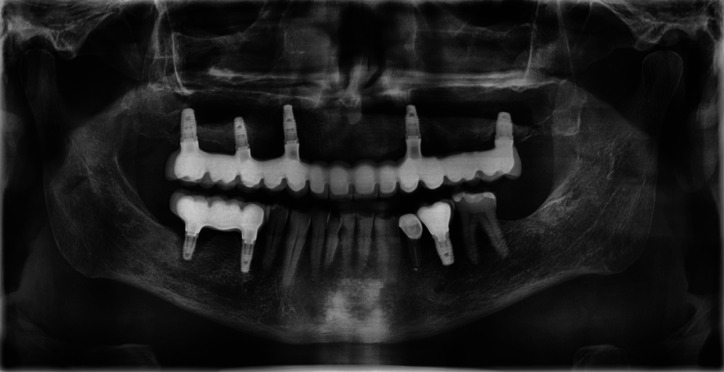
Orthopantomography carried out after one year of follow-up in which the patient showed no symptoms of sinus infection.

Male patient, 54 years old, with no medical history of interest or known harmful habits. He visits the clinic to restore the maxillary arch, in which there were some teeth remaining that were going to be removed and replaced by an implant-supported fixed prosthesis on 6 implants in positions #3, #5, #7, #10, #13 and #14. A prophylactic perioperative antibiotic treatment was administered with Amoxycillin/clavulanic acid 875/125 mg/ 8h for 7 days, starting two days before the surgery. Mouth rinse with chlorhexidine digluconate at 0.20% every 8h for 15 days was indicated. In the 3rd sextant, a sinus lift was performed with a lateral window approach (Caldwell-Luc type) due to a remaining bone height of less than 4 mm. The sinus cavity was filled with Gen-Os® (OsteoBiol), a porcine origin xenograft, with particles of cancellous bone and collagenous cortical bone with a particle size of 250-1,000 µm and a Biomet 3i implant (Zimmer®) of 5x10 mm was placed simultaneously in the position corresponding to the #13. The antrostomy was sealed with a 0.5 mm thickness collagen barrier membrane (Evolution® Standard, OsteoBiol) of porcine origin. The patient’s complete prosthesis was relined after surgery with a soft relining material. After the healing period (4 months) a second stage implant surgery was carried out to place the healing abutments, before the prosthetic phase, at which time, the implant in position #13 migrated to the sinus. Because the implant migrated to the tuberosity area in the sinus cavity and because the antrostomy had been performed in a more mesial position, the patient was seen once a week, recommending him to sleep on his right side and was prescribed ciprofloxacin 500 mg/ 12 h for 10 days. During this time, he reported nasal congestion and headache. After one week, with the implant in a more favourable position, a mucoperiosteal flap was raised and the sinus was accessed through the original antrostomy. The Schneider membrane was removed at this level and the purulent accumulation formed after the implant migration was drained. The healing process went on without any incidents and it was decided not to insert a new replacement implant, redesigning the implant-supported rehabilitation on 5 implants. During the following year of follow-up, the patient did not manifest any type of sinus symptomatology, (Figs. 1-3).

## Discussion

Depending on when it occurs, implant migration into the maxillary sinus is classified as early or late displacement. Early displacement can happen at the time of surgery due to an incorrect surgical technique (3), excessive force during implant placement (5), surgical inexperience (9), a poor or a lack of primary stability at the time of implant placement (3,9), inadequate bone quality or quantity (3), autoimmune reaction, or changes in intranasal sinus pressure (2). Late displacement may occur after implant loading due to early loading (9), inflammatory reactions that cause peri-implantitis and bone loss or implant overload, among others (3). In this case, the reason for the displacement was related to micro-movements in the implant caused by the complete removable prosthesis used as a provisional solution.

DID into the maxillary sinus can act as foreign body causing serious complications, so that, it should be removed as soon as possible, even in asymptomatic patients, to prevent the spread of infection to paranasal sinuses or other vital sites (1,7). The timing of surgical intervention should be as close as possible to the foreign body insertion to minimise mucosal inflammation and to prevent discrepancies in the location of the implant (6). The explanations of how implants migrate inside the maxillary sinus have not been clearly defined. It could be explained if it is assumed that the implant breaks through the natural maxillary sinus ostium with the aid of the mucociliary action so that the displaced implant can be accidentally swallowed or aspirated, which may be life-threatening (8). These complications are rare; however, it can be a cause of odontogenic sinusitis (10) because it can cause impairment of the mucociliary clearance mechanism (2) or tissue reaction (5). The most significant sequela due to foreign bodies in the maxillary sinus is chronic sinusitis with or without nasal polyps, which may cause serious conditions such as pansinusitis, panophthalmitis, and orbital cellulitis (1).

Lack of bone and lack of osseointegration due to insufficient quantity of vital bone may lead to implant dislocation, sinus infection and oroantral fistula formation. A sinus floor augmentation in a first stage and a delayed dental implant placement may prevent this complication by increasing the quantity and quality of bone before dental implant placement (7). Another preventive measure is placing implants in areas with sufficient available vital bone through title implants, all-on-4 techniques (in case of full-mouth rehabilitation) avoiding the maxillary sinus, or placing implants in anatomical buttresses, as well as ultra-short implants, subperiosteal dental implants or bone augmentation procedures.

Surgical strategies for the removal of a migrated implant are essentially divided into two main approaches: transoral and transnasal endoscopic approach (or combined). Oral and maxillofacial surgeons are more familiar with the transoral approach, whereas the transnasal endoscopic approach is more prone to be done by otolaryngologists. In the present case, the sinus was re-accessed through the antrostomy created during the sinus lift procedure carried out a week before as it was considered to be the least invasive procedure. In these cases, the patient can be asked to move his head in different directions to facilitate the implant migration near the lateral window. A fibre-optic light can be used to locate the implant. After dental implant removal, the sinus window will be covered with a resorbable barrier membrane. This technique (transoral approach) enables improved access and visibility for the removal of the displaced implant (3) and enables removal of all the granulation tissue due to a direct visual approach, as well as a rapid recovery of the patient´s symptoms (5).

## Conclusions

In the case of intraoperative migration of the implant into the sinus, its removal is recommended as soon as possible to avoid a possible sinus pathology of iatrogenic origin or even a complication that implies a life-threatening condition.
